# Molecular Characterization of Tob1 in Muscle Development in Pigs

**DOI:** 10.3390/ijms12074315

**Published:** 2011-07-04

**Authors:** Jing Yuan, Ji-Yue Cao, Zhong-Lin Tang, Ning Wang, Kui Li

**Affiliations:** 1 College of Veterinary Medicine, Huazhong Agricultural University, Wuhan, Hubei 430070, China; E-Mail: yuanjing@mail.hzau.edu.cn; 2 Key Laboratory for Farm Animal Genetic Resources and Utilization of Ministry of Agriculture of China, Institute of Animal Science, Chinese Academy of Agricultural Sciences, Beijing 100193, China; E-Mail: kuili@iascaas.net.cn; 3 College of Animal Science, Northeast Agricultural University, Haerbin, Helongjiang 150030, China; E-Mail: ningwang2001@yahoo.com

**Keywords:** pig, *Tob1*, muscle development, expression profile, chromosome mapping, subcellular localization

## Abstract

Cell proliferation is an important biological process during myogenesis. *Tob1* encoded a member of the Tob/BTG family of anti-proliferative proteins. Our previous LongSAGE (Long Serial Analysis of Gene Expression) analysis suggested that *Tob1* was differentially expressed during prenatal skeletal muscle development. In this study, we isolated and characterized the swine *Tob1* gene. Subsequently, we examined *Tob1* chromosome assignment, subcellular localization and dynamic expression profile in prenatal skeletal muscle (33, 65 and 90 days post-conception, dpc) from Landrace (lean-type) and Tongcheng pigs (obese-type). The *Tob1* gene was mapped to pig chromosome 12 (SSC12). The *Tob1* protein was distributed throughout the nucleus and cytoplasm of PK15 cells. During prenatal skeletal muscle development, *Tob1* was up-regulated and highly expressed in skeletal muscle at 90 dpc in Tongcheng pigs but peaked at 65 dpc in Landrace pigs. This result suggested that there were different proliferation patterns during myogenesis between Tongcheng and Landrace pigs. During postnatal skeletal muscle development, the expression of *Tob1* increased with aging, indicating that the proliferation potential of myoblasts decreased in postnatal muscle development. In tissues of adult wuzhishan miniature pigs, the *Tob1* gene was highly expressed in skeletal muscle. The expression of *Tob1* was significantly increased at day 6 during C2C12 differentiation time, suggesting a possible role in skeletal muscle development. Therefore, this study indicated that *Tob1* perhaps played an important role in skeletal muscle development.

## 1. Introduction

*Tob1* (transducer of *ERBB2*, *1*) is a member of the Tob/BTG gene family that had the potential to regulate cell growth. The Tob/BTG gene family consists of at least six members in vertebrates: *BTG1*, *BTG2*/*TIS21*/*PC3*, *BTG3*/*ANA*, *PC3B*, *Tob1* and *Tob2* [1]. The Tob/BTG proteins have a highly conserved 110-amino acid N-terminal region, designated the Tob/BTG homology domain, considered as the domain responsible for their anti-proliferative effects [2], while their C-terminal regions were necessary and sufficient to regulate the stabilities of BTG1, BTG2, Tob1, and Tob2 proteins [3].

The Tob/BTG genes are involved in cell growth (anti-proliferation) and differentiation. It had been previously reported that the *BTG2* and *BTG3* genes were associated with myoblast proliferation [4]. Tob1 was first isolated as a protein associated with the ErbB2 growth factor receptor [5]. The anti-proliferative function of Tob1 was negatively regulated through phosphorylation by extracellular signal-regulated protein kinase (Erk) 1 and 2 [6]. Exogenously expressed *Tob1* could control cell growth, and inhibited the proliferation effect to stimulate growth through its interaction with p185erbB2 [5]. *Tob1* inhibited T cell activation, blocked cell cycle [7], repressed transcription of cytokines and cyclins and was a substrate of the MAPK (mitogen-activated protein kinases) pathway [8]. The *Tob1* gene was expressed in various segments of the brain and may be involved in learning and memory in mammals [9]. Tob1 also negatively regulated osteoblast proliferation and differentiation by inhibiting the activity of the receptor-regulated Smad proteins [10,11]. Most research on *Tob1* had focused on its role in cancer, *Tob1* perhaps is an important tumor suppressor, as mice lacking the *Tob1* gene had been reported to be more prone to cancer than wide-type mice [12] and *Tob1* had lower expression levels in lung cancer tissue than in adjacent normal lung tissues in humans [12,13]. *Tob1* was expressed maternally and continuously throughout embryonic development period [14], Over-expression of *Tob1* in zebrafish embryos resulted in ventralized phenotypes, while *Tob1* knockdown led to embryonic dorsalization [15], which suggested that *Tob1* played an important role during embryonic development. These observations indicated the importance of *Tob1* in many biological processes.

Our previous LongSAGE analysis (LongSAGE was an adaptation of the SAGE approach that allows 21 bp tags to be obtained from individual transcripts) suggested that *Tob1* was differentially expressed during the development of fetal skeletal muscle [16], but there were no reports on the biological role of *Tob1* in skeletal muscle development. To understand the biological function of *Tob1* during myogenesis, we isolated and characterized the *Tob1* gene in swine.

## 2. Results and Discussion

### 2.1. Tob1 mRNA Sequences Analysis

**Figure 1:**
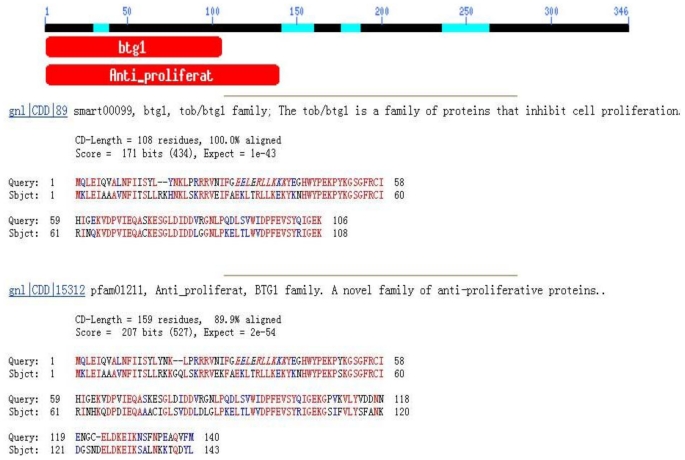
The conserved functional domains of the swine Tob1 protein.

The full-length mRNA of swine *Tob1* contained a 1041-bp open reading frame encoding a 346-amino acid protein with a predicted molecular weight of 38.257 kDa and an isoelectric point of 6.45. The mature mRNA sequence contained a 5′-untranslated region (5′ UTR) of 401 bp and a 3′ UTR of 774 bp with an AATAAA polyadenylation signal. The obtained sequence had 60 bp using the GLGI method. The isolated gene sequence was submitted to Genebank (Genebank No.: EF486515). The swine *Tob1* gene sequence had 94% similarity with the human *Tob1* gene. We predicted the conserved domain from the deduced amino acid sequence using BlastP. Morever, the RPS-blast program predicted that swine Tob1 amino acids 1–118 and 1–140 contained typical BTG1 and anti-proliferative conserved domains, respectively (Figure 1), suggesting that this protein was a member of the BTG family.

### 2.2. Temporal and Spatial Expression

**Figure 2:**
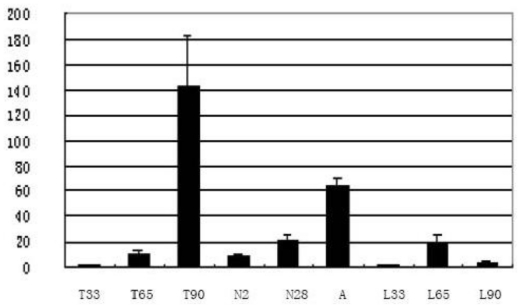
The expression profile of *Tob1* in longissimus dorsi muscle from Tongcheng and Landrace pigs at different development stages by real time PCR. T33, T65, T90, N2, N28, A represented longissimus dorsi muscle from Tongcheng pigs at 33, 65, 90dpc, at postnatal 2, 28 days and adult periods, respectively. L33, L65, L90 represented longissimus dorsi muscle from Landrace pigs at 33, 65, 90 dpc, respectively.

While *Tob1* had been reported to be expressed in skeletal muscle of mice [17] and humans [5], the expression pattern of *Tob1* during the development of skeletal muscle has not been reported for swine. This study analyzed the expression level of *Tob1* in skeletal muscle at different developmental stages and found that the swine *Tob1* gene had different expression levels at various stages of skeletal muscle development in different pig breeds. Both LongSAGE (Table 1) and the quantitative PCR (Figure 2) analysis suggested that the *Tob1* gene peaked at 90 dpc in Tongcheng pigs and 65 dpc in Landrace pigs during prenatal skeletal muscle development. The *Tob1* gene was differentially expressed in skeletal muscle in Tongcheng and Landrace fetal pigs, indicating that the gene perhaps involved in the phenotypic differences in muscle fiber between Chinese and foreign breeds of pigs. The function of *Tob1* gene was anti-proliferation, thus expression difference perhaps was one of the reasons that associated to the birth weight difference of Tongcheng and Landrace pigs. In postnatal skeletal muscle in Tongcheng pigs, we found that *Tob1* was up-regulated with increased age (Figure 2) which suggested that the *Tob1* gene was related to the development of skeletal muscle.

**Figure 3:**
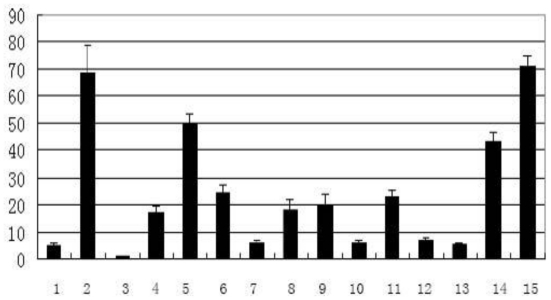
The expression profile of *Tob1* in different tissues of adult pigs. The tissues examined were (1) lung; (2) biceps femoris; (3) spleen; (4) heart; (5) stomach; (6) large intestine; (7) lymph; (8) small intestine; (9) liver; (10) brain; (11) longissimus dorsi (LD); (12) kidney; (13) fat; (14) gastrocnemius; and (15) semitendinosus. The values shown are Mean ± SD levels of *Tob1* from three independent experiments. The level of *Tob1* in spleen was arbitrarily set to 1.0.

The tissue distribution of *Tob1* mRNA suggested that it was highly expressed in skeletal muscle, heart, and liver and weakly expressed in spleen, lung, fat tissues of adult pigs (Figure 3). The human *Tob1* gene is highly expressed in heart, liver and kidney but weakly expressed in lung, fat and spleen using the UCSC Genome Bioinformatics [18]; thus, *Tob1* exhibited similar tissue expression patterns between pigs and humans.

### 2.3. Tob1 Expression During C2C12 Differentiation Time

**Figure 4:**
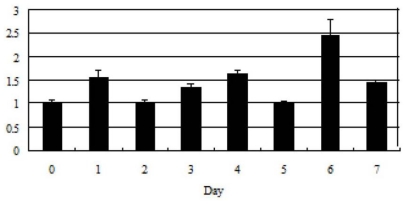
Expression pattern of the mouse *Tob1* gene during C2C12 differentiation time. The values were normalized to GAPDH mRNA expression level. Day 0 expression level was set to 1. The error bars indicate Mean ± SD (n = 3). 0: C2C12 myoblast cells; 1–7: days 1–7 of myoblast differentiation into myotubes.

We investigated the expression of *Tob1* during C2C12 myoblast differentiation time using Real-time PCR with GAPDH as an internal control. The statistics were Student’s *t*-test of 1–7 day during differentiation time compared to 0 day. The expression of *Tob1* was significantly increased at day 6 during C2C12 differentiation time (*p* < 0.05), suggesting its role in skeletal muscle development (Figure 4). This further suggested that *Tob1* was perhaps involved in myoblast differentiation.

### 2.4. Chromosome Mapping Using IMpRH

Using the INRA-University of Minnesota porcine radiation hybrid panel ( IMpRH panel), we found that the swine *Tob1* gene was closely linked (two-point analysis) to the microsatellite marker SS04H11 on pig chromosome 12 (SSC12). The SS04H11 marker was mapped to 12p11-p13; therefore, the most likely chromosomal location for *Tob1* was assigned as 12p11-p13 (Table 2). Pig chromosome 12 accounted for 3.5% of the pig genome, corresponded to human chromosome 17. The human *Tob1* gene was mapped to chromosome 17 (SSC17), which was in agreement with the above conclusion.

### 2.5. Subcellular Localization of Tob1 in PK15 Cells

**Figure 5:**
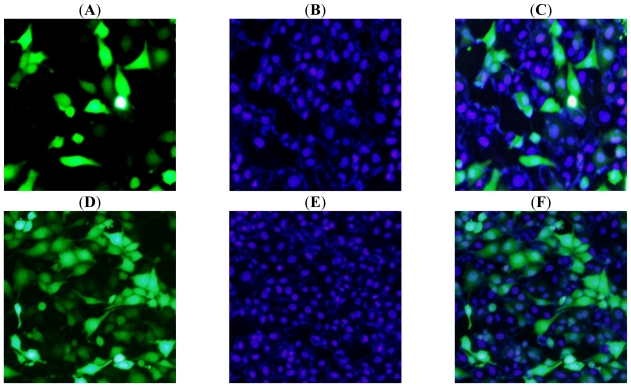
Subcellular localization of the pEGFP-Tob1 fusion protein in PK15 cells. **(A)** Distribution of fluorescence after transfection of the pEGFP-Tob1 vector; **(B)** PK15 nuclei stained with Hoechst 33342; **(C)** The merged image of A and B; **(D)** GFP detected in PK15 cells transfected by the pEGFP-N3 empty vector; **(E)** Nuclei of PK15 cells transfected by the pEGFP-N3 empty vector; **(F)** The merged image of D and E.

To determine the subcellular localization of Tob1, we transfected pEGFP-Tob1 plasmid into PK15 cells. PSORT II program analysis predicted that Tob1 may be localized primarily to the nucleus (65.2% probability), with lower probabilities found for the cytoplasm (8.7%), plasma membrane (4.3%). But our analysis of pEGFP-Tob1 fusion protein indicated that the pEGFP-Tob1 fusion protein was distributed throughout the nucleus and cytoplasm of the PK15 cells using fluorescence microscopy. Green fluorescence was detected through control cells transfected with GFP vector alone (Figure 5).

In human Tob1, Tob1 was shuttling between the nucleus and the cytoplasm by its nuclear localization signal (NLS) and nuclear export signals (NES) [19], NLS and NES played important roles in subellular distribution [19,20]. When the cells stayed at G0 phase, Tob1 was mainly localized in the nucleus. Until early G1 phase, most of Tob1 protein still localized in the nucleus, but when the cells entered into the late S phase, more than half of Tob1 protein was detected in the cytoplasm [18], which suggested that the subcellular distribution of Tob1 also varied with cell cycle. It seemed that mechanisms of nuclear *versus* cytoplasmic localization of Tob1 protein will have some effects on its anti-proliferative function.

## 3. Experimental Section

### 3.1. Source of Animals and Tissues

Pig embryos or fetuses were collected from pregnant Tongcheng and Landrace pigs during three embryonic periods (33, 65 and 90 days post-conception, dpc) and three postnatal periods (day 2, day 28 and adults period). The longissimus dorsi (LD) muscles were isolated. The different fifteen tissues were acquired from mature Wuzhishan miniature pigs for expression pattern analysis. All the tissue samples were collected from three individual pigs at each period. All the tissues were harvested, frozen in liquid nitrogen, and stored at −80 °C until use.

### 3.2. RNA Extraction and cDNA Preparation

Total RNA was isolated using Trizol reagent (Invitrogen, Carlsbad, CA, USA), according to the manufacturer’s instructions. RNA measurement was conducted using Evolution 60 UV-Visible spectrophotometer (Thermo Fisher Scientific Inc, Portsmouth, NH, USA) by measuring ultraviolet absorbance at 260 nm and 280 nm. Calculation of the RNA concentration was based on the absorbance at 260 nm. Furthermore, RNA purity was estimated as the 260 nm/280 nm ratio, and the RNA samples were then stored at −80 °C.

The cDNAs were reverse-transcribed from RNAs using an oligo(dT) primer and M-MLV reverse transcriptase (Promega, Madison, WI, USA). The conditions were as follows: 5 min at 37 °C followed by 60 min at 42 °C and 10 min at 72 °C. The resulting cDNA samples were stored at −20 °C.

### 3.3. Cloning, Sequencing and Analysis of Tob1 mRNA Sequences

A BLAST search was performed to obtain pig ESTs based on the human *Tob1* mRNA sequence (GenBank: NM_005749.2), and mRNA sequence of the swine *Tob1* gene was obtained by assembling ESTs with more than 80% identity to the human mRNA sequence. The partial mRNA sequence of swine *Tob1* was amplified with Tob1F, R primers (Table 3) using skeletal muscle cDNA as template. The PCR product was purified and subsequently cloned into the pGEM T-easy vector (Takara, Otsu, Japan) and sequenced using M13-forward and M13-reverse primers.

The protein domain analysis was performed using PSORT II [21] and PROSITE [22].

### 3.4. Isolation of 3′ cDNA by GLGI

To identify the tag sequence and obtain the 3′ UTR sequence of the swine *Tob1* gene, the LongSAGE tag of 17 bp (CAGTATTCTAACCAGCA) was changed into its corresponding 3′ UTR sequence using the GLGI method (the generation of longer cDNA fragments from SAGE tags by converting novel SAGE tags into 3′ cDNAs for gene identification, GLGI) [23]. The GLGI experiment was carried out as previously reported [16,24]. The PCR product was sequenced and aligned for gene sequence identification.

### 3.5. TaqMan Real-Time PCR Analysis

We analyzed the dynamic expression profile in prenatal skeletal muscle (33, 65 and 90 days post-conception, dpc) from Landrace and Tongcheng pigs. The expression of *Tob1* in postnatal skeletal muscle (2 day, 28 day and adults) from Tongcheng pigs and all kinds of tissues in adult wuzhishan miniature pigs were also examined using TaqMan real-time PCR.

The TaqMan real-time PCR system contained 10 × PCR buffer (Takara, Otsu, Japan), 3.0 mM MgCl_2_, 100 μM each dNTP, 0.3 μM primers, 0.1 μM probe, 2 U Taq DNA polymerase (Takara, Otsu, Japan), 2 μL cDNA template, add ultrapure water to a total volume of 20 μL. The cycling conditions consisted of an initial cycle of 5 min at 95 °C followed by 35 cycles of 15 s at 95 °C and 1 min at 60 °C. The PCR reactions were performed in biological triplicate for each sample. The gene expression levels were quantified relative to the expression of GAPDH using Gene Expression Micro (Bio-Rad, Richmond, VA, USA) employing the comparative cycle threshold (ΔΔCt) method [25].

### 3.6. Real-Time PCR Analysis During C2C12 Differentiation Time

C2C12 established from normal adult C3H mouse leg muscle, was good model to study myogenesis and cell differentiation *in vitro*.

The cells were cultured in Dulbecco’s modified Eagle’s medium (DMEM) supplemented with 10% (v/v) FBS, 2 mM glutamine, 100 U/mL penicillin and 100 μg/mL streptomycin and maintained at 37 °C in 5% CO_2_. To induce myogenic differentiation, C2C12 cells were seeded in six-well plates. After about 12–16 h, when cell confluence reached approximately 60–70%, the differentiation of C2C12 myoblasts into myotubes was induced by the addition of differentiation medium (DMEM contained 2% horse serum instead of 10% FBS). We collected the cells for RNA extraction and H.E staining every day during C2C12 differentiation time. The myotubes were confimed by H.E staining in morphological level and creatine kinase muscle (CKM) marker in biochemical characterization.

The Real-time PCR system contained 50 × ROX Reference Dye II, 2 × SYBR Green Real-Time PCR Master Mix (Takara, Otsu, Japan), 10 μM primers and 1 μL cDNA template in a total volume of 20 μL. The cycling conditions consisted of an initial step of 15 s at 95 °C followed by 40 cycles of 5 s at 95 °C and 34 s at 62 °C. The PCR reactions were performed in an ABI 7500 FAST Real-time PCR instrument in biological triplicate for each sample. The gene expression levels were normalized to GAPDH as described above.

### 3.7. Chromosome Mapping Using IMpRH

The chromosome assignment was performed using the INRA-University of Minnesota 7000-rad radiation hybrid panel (IMpRH) consisting of 118 hamster-swine hybrid cell lines [26]. The PCR reaction was carried out in a total volume of 10 μL containing 10 × PCR buffer (Mg^2+^, Fermentas, Burlington, ON, Canada), 75 μM each dNTP, 0.3 μM each primer, 25 ng panel DNA, and 1.0 U Taq DNA polymerase (Fermentas, Burlington, ON, Canada). The PCR conditions were 94 °C for 3 min, 35 cycles of 20 s at 94 °C, 20 s at 62 °C, and 20 s at 72 °C, and a final extension of 5 min at 72 °C. The data analysis was performed using the IMpRH [27].

### 3.8. Construction of the Subcellular Localization Vector

We used Loc-F/Loc-R primers (Table 3) to amplify the full coding sequence of the *Tob1* gene. ECO47III and BglII restriction sites were incorporated at the 5′ ends of the forward and reverse primer, respectively. After T-A cloning, the PCR fragment was double-digested using ECO47III and BglII. The product was then inserted into the linear pEGFP-N3 vector, which was digested using the same enzymes (MBI, Houston, TX, USA), to generate the pEGFP-Tob1 plasmid. Subsequently, pEGFP-Tob1 was sequenced by Invitrogen to verify the sequence of the inserted fragment.

### 3.9. Cell Culture and Transient Transfection

Pig kidney cells (PK15) were seeded in six-well plates and cultured in DMEM supplemented with 10% FBS, 4 mM glutamine, 100 U/mL penicillin and 100 U/mL streptomycin at 37 °C in 5% CO_2_.

When the cells had reached approximately 80% confluence, we performed transient transfections using 10 μL Lipofectamine 2000 reagent (Invitrogen, Carlsbad, CA, USA) with 4 μg of pEGFP-Tob1 or pEGFP-N3 plasmid DNA (Promega, Madison, WI, USA). The transfection medium was replaced with normal growth medium after 6 h. At 24 h after transfection, the cells in 6-well plate were washed three times using phosphate-buffered saline, then fixed for 15 min with 4% para-formaldehyde. After the washing steps, the cells were stained with 10 μM Hoechst 33342 for 10 min. The subcellular localization of the pEGFP-Tob1 fusion protein was determined using fluorescence microscopy.

## 4. Conclusions

In conclusion, we isolated and characterized the swine *Tob1* gene. Subsequently, we examined *Tob1* chromosome assignment, subcellular localization and dynamic expression profile in prenatal skeletal muscle. Our data indicated that the swine *Tob1* gene was differentially expressed throughout skeletal muscle development, which suggested a role for *Tob1* in the regulation of skeletal muscle development. It also exhibited different expression patterns between Tongcheng (obese-type) and Landrace (lean-type) pigs during myogenesis, the *Tob1* gene was highly expressed in skeletal muscle at 90 dpc in Tongcheng pigs and it was much higher than that in Landrace pigs. These findings suggested that the *Tob1* gene contributed to phenotypic differences in muscle between Chinese and foreign breeds of pigs and should be considered as a candidate gene for meat production traits.

Taken together, our findings will provide a basis for future study on *Tob1* function in regulation of skeletal muscle development.

## Figures and Tables

**Table 1:** Significance analysis for the differential expression of *Tob1* in the embryonic skeletal muscle LongSAGE libraries from Tongcheng and Landrace pigs at different developmental stages.

	T33	T65	T90	L33	L65	L90
T33	1 (1)	0.5058 (3)	0.0045** (11)	1 (1)	0.3232 (3)	0.4929 (2)
T65		1	0.0287*	0.3162	0.6567	0.5321
T90			1	0.0034**	0.0273*	0.0129*
L33				1	0.3384	0.4616
L65					1	0.5546
L90						1

( ) represents sequence counts of LongSAGE tag;

* indicates *p* < 0.05;

** indicates *p* < 0.01.

**Table 2:** RH mapping results for the swine *Tob1* gene.

Gene	Retention fraction%	Chromosome	Linked marker	Breakage frequency	RH distance(Ray)	LOD score
*Tob1*	27	12	SS04H11	0.5	0.69	5.52

**Table 3:** Primers and probes used in these experiments.

Primer	Sequences (5′–3′)	Size (bp)	Tm (°C)
Tob1F	AAGCAGCCCGAACAAGAC	1462	55.6
Tob1R	AATCAGCCATGTCCTTGC		
Tob1-MF	GACCCCGTCCTCGCCAAC	170	64
Tob1-MR	TGTTCGGGCTGCTTCCACC		
GLGIF	CATGCAGTATTCTAACCAGCA	60	54.4
GLGIR	ACTATCTAGAGCGGCCGCTT		
Exp-F	TGATCGAGCAGGCATCCAA	116	58
Exp-R	TTCGCCGATCTGGTAGGAAAC		
GAPDH-F	GGTGAAGGTCGGAGTGAACG	233	60
GAPDH-R	CTCGCTCCTGGAAGATGGTG		
Loc-F	**AGCGCT**ATGCAGCTTGAAATCCAAGTAG	1038	61
Loc-R	**AGATCT**GTTAGCCATAACAGGCTGGAAT		

Tob1F, R: primers used to amplify the partial mRNA sequence; Tob1-MF, MR: primers used to analyze the expression pattern of swine *Tob1*; GLGIF, R: primers used to amplify the 3′ end of the *Tob1* cDNA sequence; Exp-F, R: primers used to analyze the expression level of mouse *Tob1* in C2C12 cells; GAPDH-F, R: primers used as an internal control; Loc-F, R: primers used to construct the expression vector pEGFP-Tob1.
